# Research on Lightweight Rose Disease Detection Based on Transferable Feature Representation

**DOI:** 10.3390/plants15040623

**Published:** 2026-02-16

**Authors:** Li Liu, Tao Yin, Yuyan Bai, Bingjie Yang, Jianping Yang

**Affiliations:** College of Big Data, Yunnan Agricultural University, Kunming 650201, China; 2023033@ynau.edu.cn (L.L.);

**Keywords:** rose disease detection, knowledge distillation, lightweight model, feature representation transfer, precision agriculture

## Abstract

Rose leaf diseases severely reduce yield and product quality, and traditional disease monitoring relies on manual visual inspection by experts, which is inefficient for large-scale cultivation. However, deploying accurate and lightweight detectors in field environments remains challenging due to two main obstacles. First, models trained under controlled laboratory conditions suffer performance degradation due to domain shift when deployed in complex field environments. Second, the computational capacity of hardware deployable in the field is often limited. To address these problems, this study proposes a practical knowledge distillation approach based on transferable feature representations from a pre-trained teacher model, rather than on complex distillation architecture. A high-capacity YOLOv12-L teacher, pre-trained on laboratory images, guided the training of a compact YOLOv12-N student using field images. The distilled YOLOv12-N student model achieved an mAP@50 of 81.1% on field test set, representing a 3.5% improvement over the baseline YOLOv12-N model, while maintaining a highly efficient architecture of only 2.56 million parameters and 6.3 GFLOPs. Several ablation studies confirm the core contribution of this work, namely that the performance gains in lightweight detection stem primarily from the transfer of the teacher model’s feature representations, rather than from modifications to the distillation algorithm or student model’s architecture, thus clarifying the importance of high quality feature transfer in cross-domain agricultural vision tasks. This approach provides a generalizable and efficient solution for real-time rose leaf disease detection in precision agriculture.

## 1. Introduction

Rose cultivation has ornamental, economic and ecological value worldwide. In China, Yunnan Province is the major production region where rose is a signature crop and contributes greatly to local agriculture and exports [1,2]. However, rose productivity and quality are threatened by foliar diseases, chiefly black spot, powdery mildew, and downy mildew [3,4,5,6]. These pathogens thrive in the warm, humid climate of southern China and can cause severe yield and quality losses [7]. Timely disease detection is therefore necessary to reduce the impact. Traditional monitoring depends on manual visual inspection by experts, which is labor intensive and subjective, and it is difficult to monitor large scale cultivation or early lesions [7,8]. Automated real-time diagnostic systems that work reliably in real-world field conditions are needed for sustainable rose production.

Automated detection approaches have evolved from machine learning methods, such as SVM or KNN [9,10,11], to deep learning approaches. Convolutional Neural Networks (CNNs) have proven to have excellent performance in crop disease classification under controlled conditions. For example, a modified AlexNet with multi-scale convolutions and dilated convolutions had scored 98.62% accuracy on maize disease detection [12]. A CNN-based integrated model for classification in complex backgrounds achieved 93.75% average accuracy and 100% recall [13]. GoogleNet achieved 99.94% accuracy in northern corn leaf blight detection [14]. However, many high-performance CNN architectures have computational costs and parameter counts, which limit direct field deployment [15].

One-stage object detectors are preferred for real-time detection of crop diseases in agricultural. The YOLO series is preferred for its efficiency [16,17]. YOLO architectures have been refined in the recent years to address crop disease detection problems such as small lesion size, symptom variability, and field deploying problems. For instance, Zhao et al. developed an enhanced YOLOv7 model with improved multi-scale feature fusion and attention functions for crop disease detection [18]. A customized YOLOv8 model with integrated attention modules for onion foliar disease detection was proposed by Raja et al. [19]. Wang et al. introduced the TomatoGuard-YOLO framework based on an improved YOLOv10 architecture [20]. However, the application of the recent iteration, YOLOv12, remains largely unexplored for plant disease detection, particularly for special plants such as rose. In addition, deploying even advanced models directly in the field faces two persistent challenges. First, field environments introduce significant domain shifts, including variable illumination, complex backgrounds and motion blur, which degrade model generalization [21,22,23]. Second, practical deployment requires lightweight models running on resource-constrained devices such as drones and field-deployed portable sensors [24,25,26].

Knowledge distillation (KD) has emerged as a promising method to bridge this gap by transferring knowledge from a large accurate teacher model to a small student model [27,28]. Li et al. demonstrated that KD improves the efficiency of the YOLOX detector under constrained resources [29]. Combining KD with customized YOLO architectures has enabled progress in crop monitoring tasks. Hu et al. applied channel-wise distillation in an improved YOLOv5s for maize leaf disease detection [30]; Yang et al. incorporated an adaptive weighted KD strategy into a redesigned YOLOv7-tiny (MFD-YOLO) for real-time strawberry growth stage monitoring [31]; and Yang et al. used KD to optimize a YOLOv11 model (StomaYOLO) for precise detection of microscopic stomata in maize leaves [32]. Further, specialized distillation strategies have been developed for specific challenges in object detection. Zheng et al. proposed Localization Distillation (LD) to transfer location-aware knowledge to improve bounding box accuracy for small or overlapping objects [33].

One of the main focuses of recent KD work is to refine distillation by means of loss functions or feature alignment [34,35,36]. This focus potentially overlooks the fundamental question regarding the nature of transferable knowledge in the context of domain shifts. The importance of teacher model feature quality as opposed to distillation design is not explored in agricultural vision. For rose leaf disease detection confronted with a lab-to-field domain shift, it is posited that the key problem is the transfer of robust features. It is demonstrated that a standard KD based on a well-pre-trained teacher model is sufficient for significant performance gains. A key pseudo-labeling experiment shows that a student trained only on the teacher’s hard decisions performs like a student trained using full KD indicating that the key to successful adaptation lies in the teacher’s feature space.

In summary, this work shifts the focus in knowledge distillation from the design of the distillation mechanism to the quality of transferred knowledge. It demonstrates that feature representations from high-quality teacher models are more important than distillation design for lightweight field deployment. This leads to a practical detection framework using a multi-component distillation design to bridge the gap between laboratory and field conditions. Its main mechanism is a powerful teacher trained in laboratory data guiding a small student trained in limited field data. Thus, this can be used as a feasible solution of precision agriculture balancing detection accuracy and computational cost of field deployment.

## 2. Materials and Methods

### 2.1. Dataset and Data Augmentation

Two publicly available datasets from the Mendeley Data platform were used in this study. A lab-to-field domain adaptation framework was established to address the paucity of high-quality annotated field-collected data for rose leaf disease detection. Key dataset characteristics are summarized in Table 1.

#### 2.1.1. Dataset Sources

1. Laboratory dataset

The lab dataset came from the ‘Multifaceted Rose Leaf Disease Dataset for AI-Driven Plant Pathology’ dataset. The images are of high quality, captured under consistent illumination. To ensure the teacher model would learn lesion-specific features without background bias, a subset of images with homogeneous backgrounds was selected. For black spot, downy mildew, and healthy leaves, the images with pure backgrounds were selected. For powdery mildew, the original dataset lacked uniform background images. Leaf segmentation was performed and the background was replaced by a same color. Manual background homogenization may lead to bias, because uniform background training could bias the model to different background textures in real-world environments. However, this trade-off can help the teacher model focus on disease-specific visual patterns such as the fine powdery texture of powdery mildew instead of background information. Further distillation with field data helps to counteract bias by aligning teacher feature knowledge with real-world situations. The final lab set contains 2000 images, with a balanced distribution across the four classes.

2. Field dataset

The ‘Cut-flower Disease Dataset’ is a realistic and challenging scenario for rose disease detection. Images were taken in natural environments with cluttered backgrounds like soil and weeds, variable lighting, and uncontrolled camera angles. Rose black spot produces irregular spreading lesions that blend with background clutter, powdery mildew creates white powdery layer that can be confused for dust or specular highlights, and downy mildew produces pale diffuse lesions that merge with healthy tissue under changing light.

**Figure 1:**
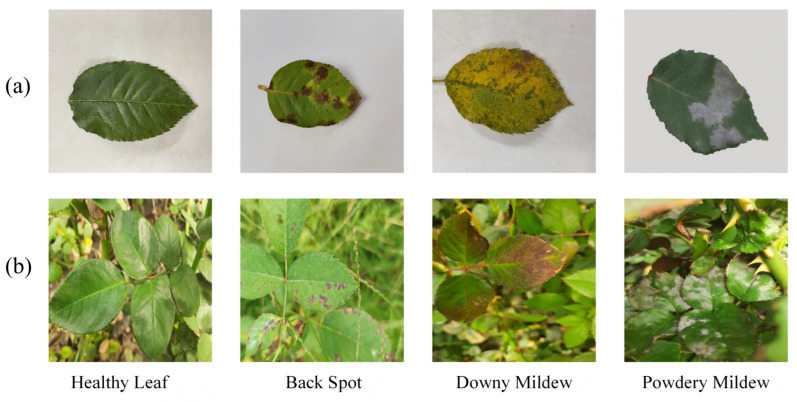
Sample images from (**a**) laboratory dataset and (**b**) field dataset.

Consequently, feature distributions differ substantially between field and laboratory samples. Sample images from both datasets are shown in Figure 1. Panel (a) presents representative laboratory images with clean, uniform backgrounds showing different disease symptoms. Panel (b) shows field images with cluttered backgrounds, variable lighting, and ambiguous lesion appearances.

#### 2.1.2. Data Augmentation

1. Laboratory dataset

During the training of the teacher model, a conservative augmentation strategy was adopted. Only mild online augmentations were applied, including random horizontal flipping with a probability of 0.5, and small brightness and contrast adjustments within ±15%. These limited modifications prevent artificial distortions and help teacher keep accurate disease symptoms. Since the augmentations were performed online during training, the lab dataset size remained the same.

2. Field dataset

The original field dataset comprised 990 images, which were partitioned into an independent training set (792 images), a validation set and a test set with no mutual overlap between all subsets. A hybrid augmentation pipeline was developed, and all augmentation operations were exclusively applied to the training set to avoid data leakage. The online stage included random horizontal flipping (probability 0.5) and color jitter (hsv_h = 0.02, hsv_s = 0.4, hsv_v = 0.2). Color jitter was used to simulate variable field lighting. Spatial transforms (translate = 0.1, scale = 0.2) were also included to account for different shooting angles. The offline stage produced two variants of training images per original training image combining flipping, stronger brightness adjustments of 20% and additive Gaussian noise (σ = 0.01). This pipeline increased field training set from 792 to 2376 images and improved student model robustness to field noise.

### 2.2. Experimental Configuration and Training Details

#### 2.2.1. Hardware and Software Environment

All experiments were conducted on a server equipped with an NVIDIA GeForce RTX 4090 GPU (24 GB VRAM), using Python 3.10.19, PyTorch 2.3.1 (CUDA 11.8), and Ultralytics 8.3.220.

#### 2.2.2. Unified Training Protocol

All model variants were trained under identical conditions for fair comparison:

- Training epochs: 200; input resolution: 640 × 640; batch size: 8. A moderate batch size of 8 was chosen for high input resolution and deep model structure, ensuring training stability while avoiding gradient dissipation due to overly large batches and fitting within GPU memory constraints.
- Optimizer: SGD (momentum = 0.937, weight decay = 5 × 10−4); learning rate scheduler: cosine annealing (cos_lr = True).
- Stopping Criterion: Training stopped after 200 epochs, and early stopping was not activated. Validation loss leveled off after about 150 epochs, and no significant performance improvement was seen in the last 50 epochs, confirming model convergence.
- Reproducibility: A fixed random seed (3407) was used for all random processes to ensure experiment reproducibility.
- Augmentation constraints: Mosaic and MixUp were disabled (mosaic = 0.0, mixup = 0.0) to avoid distorting the distinct symptoms of rose diseases.

#### 2.2.3. Evaluation Metrics

The performance of the test set of the field set was measured using standard detection metrics, such as mAP@50, mAP@50-95, Precision (P), Recall (R), and F1-score. The scores of mAP@50 should be prioritized to meet the practical requirements for accurate lesion localization and identification. All experiments were repeated five times and results were presented as mean ± standard deviation (SD).

Model efficiency was measured by three model parameter count measures, namely model parameter count (M), GFLOPs at a resolution of 640 × 640, and inference speed (FPS) tested on an RTX 4090 GPU. Efficiency metrics were computed according to standard protocol for lightweight detector evaluation for agricultural vision tasks, which can be comparable with existing studies.

### 2.3. Selection of Base Detector

The YOLO family was chosen for its efficiency in real-time agricultural vision. YOLOv12 introduces several significant improvements, making it ideal for edge applications in agriculture [37]. It incorporates Area Attention (A2) module for local feature extraction, R-ELAN, to ensure smooth gradient flow and effective feature aggregation, and FlashAttention to optimize memory usage during computation [38]. The light YOLOv12-N variant achieved an exceptional balance of efficiency and accuracy. With 2.6 million parameters, it attained 40.6% mAP@50 on COCO while running at 1.64 ms inference latency. This was ahead of earlier compact models, where YOLOv10-N had 38.5% mAP@50 with 2.3M parameters and YOLOv11-N had 39.4% mAP@50 with 2.6M parameters. For more demanding detection tasks, the larger YOLOv12-L model was well-compensatory and had robustness improvement. On COCO, it reduced computational cost by 31.4 GFLOPs in comparison to YOLOv10-L and delivered 0.4% higher mAP@50 than YOLOv11-L but with same number of parameter count [38]. In an agricultural study of occlusion rich orchards, YOLOv12-L achieved the highest reported mAP@50-95 of 66.22% for multi-class green fruit detection [39]. This result demonstrates its robustness under label ambiguity and partial occlusion. In summary, both the YOLOv12-L and YOLOv12-N variants have complementary strengths. YOLOv12-L learns rich discrimination features and the YOLOv12-N learns efficient inference. This complementary design provides the basis for the proposed knowledge distillation framework.

### 2.4. Knowledge Distillation Framework

**Figure 2:**
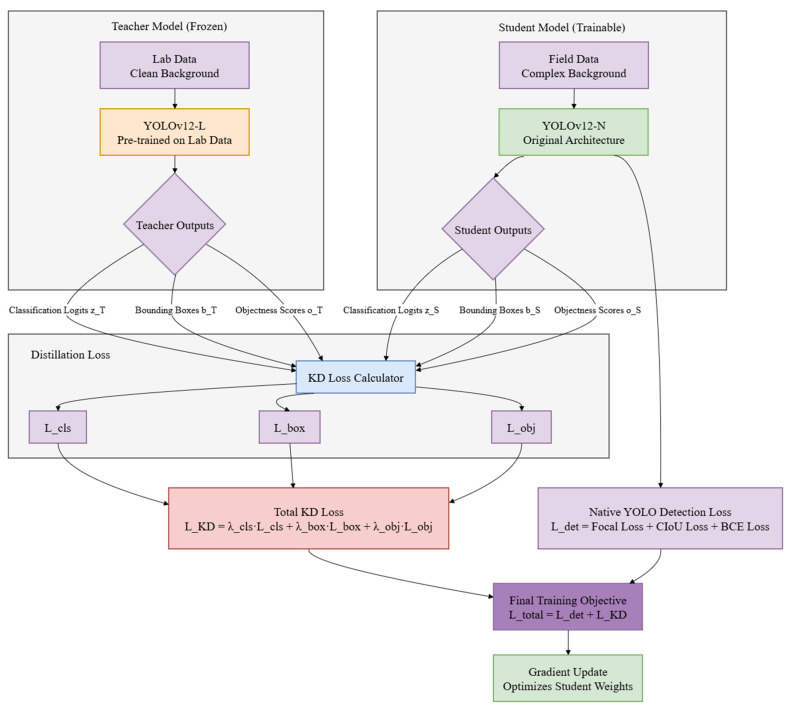
Proposed knowledge distillation framework.

This study proposes a teacher–student distillation framework tailored to address limited annotated field data and lab-to-field domain shift, as illustrated in Figure 2.

#### 2.4.1. Teacher–Student Configuration

The teacher model adopted YOLOv12-L, which was pre-trained to convergence on the laboratory dataset. Because of its high capacity, the teacher reliably encoded fine-grained features of rose diseases, for example the subtle texture differences between powdery and downy mildew and the irregular shapes of black spot lesions. The teacher remained frozen during distillation and thus provided stable, robust feature representations and soft labels.

For the student model, the lightweight YOLOv12-N was selected. The student was trained only on the augmented field dataset while receiving knowledge transferred from the frozen teacher. This distillation may reduce domain shift that arises from the discrepancy between the clear, consistent symptoms in laboratory images and the ambiguous, cluttered disease manifestations in real-world field environments.

#### 2.4.2. Multi-Component Distillation Loss Design

A multi-component distillation loss was formulated to address specific challenges posed by the visual characteristics of rose diseases.

1. Classification Distillation

Powdery mildew and downy mildew both appear as surface deposits in the field, which creates classification ambiguity. To convey subtle textural differences, softened probabilistic knowledge was transferred by means of the Kullback–Leibler divergence with temperature scaling [40], as shown in Equation (1):

(1) $$L_{\mathrm{cls}}{=\tau }^{2}\cdot \mathrm{KL}\left(\sigma \left(\frac{z_{T}}{\tau }\right)∥\sigma \left(\frac{z_{S}}{\tau }\right)\right)$$

where $z_{T}$ and $z_{S}$ are teacher and student class logits, respectively, τ is the temperature parameter, and σ denotes the softmax function. This allows the student to learn from the relative probability distributions of the teacher and make more precise decision boundaries for these confusable classes. The default value of the temperature parameter τ was 4 because it is used widely in knowledge distillation and has shown proven efficacy in prior studies [41].

2. Bounding Box Distillation

Black spot lesions have highly irregular, spreading edges, and early-stage lesions across categories are often small and dispersed; therefore, precise localization is essential for severity assessment. An L1 loss was applied to the bounding box parameters to produce stable, direct regression of box coordinates, as shown in Equation (2):

(2) $$L_{\mathrm{box}}=\frac{1}{N}\sum_{i=1}^{N}{\left\|b_{T}^{\left(i\right)}{-b}_{S}^{\left(i\right)}\right\|}_{1}$$

where $b=(c_{x}{,c}_{y},w,h)$ represents the bounding box coordinates. The L1 norm provides consistent gradient flow, encouraging the student to precisely localize irregular and small lesions as learned from the teacher’s clear lab-based examples.

3. Objectness Confidence Distillation

Field images include background clutter such as soil patterns, leaf shadows, and other foliage that can resemble disease symptoms. Because the teacher model is trained on clean, uniform backgrounds and yields more reliable objectness signals, the student’s confidence scores were aligned to those of the teacher by means of a Mean Squared Error (MSE) loss, as shown in Equation (3):

(3) $$L_{\mathrm{obj}}=\frac{1}{N}\sum_{i=1}^{N}{{(o}_{T}^{\left(i\right)}{-o}_{S}^{\left(i\right)})}^{2}$$

where $o_{T}$ and $o_{S}$ are teacher and student objectness predictions. This component helps the student model distinguish between genuine rose disease lesions and background noise, a common challenge in dense rose planting scenarios.

The student’s total training objective combines the native YOLO detection loss with the weighted distillation terms described above. The total distillation loss can be expressed as shown in Equation (4):

(4) $$L_{\mathrm{total}}{=L}_{\det}{+\lambda }_{\mathrm{cls}}L_{\mathrm{cls}}{+\lambda }_{\mathrm{box}}L_{\mathrm{box}}{+\lambda }_{\mathrm{obj}}L_{\mathrm{obj}}$$

where $L_{\det}$ is the native YOLO detection loss, which comprises Focal Loss for classification, CIoU for bounding box regression, and BCE loss for objectness. The default weighting coefficients were set as λ_cls:λ_box:λ_obj = 1:2:1.

### 2.5. Pseudo-Labeling Experiment

**Figure 3:**
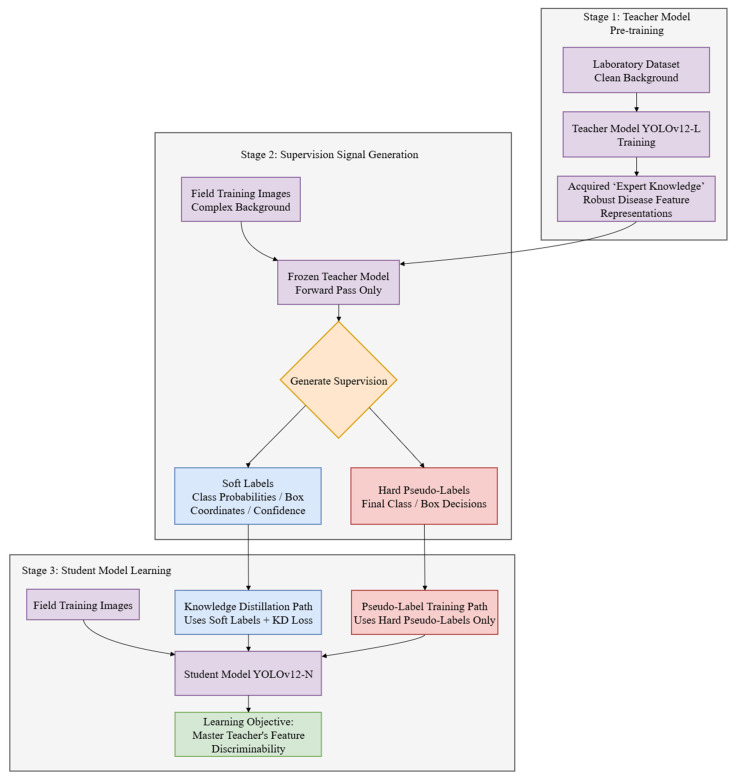
Comparison of standard KD and pseudo-label training.

To assess whether performance improvements arise from the distillation loss formulation or from the intrinsic knowledge of the teacher model, a crucial ablation study known as pseudo-label training was conducted. This experiment compared two learning methods that use the same setup and differ only in supervision type. The flowcharts of the two approaches are illustrated in Figure 3.

The established procedure using the frozen YOLOv12-L teacher model pre-trained on laboratory data was employed. A significant difference was observed in the second step of the experiment. The student model in standard knowledge distillation underwent training with the complete multi-component distillation loss, which incorporates probabilistic soft labels from teacher model. In contrast, the student model for the pseudo-label trained by hard labels that correspond to final class assignments and bounding boxes. These hard labels were generated by the teacher model via an offline forward pass executed on the field training set. All other training conditions remained identical to standard KD student, including data, enhancement pipeline, optimizer configuration and number of training epochs.

If the student model that only uses hard pseudo-labels performs similar to the student trained with complete KD loss, it would show that the crucial, transferable knowledge is present in teacher output decisions. This finding would highlight that the primary function of the distillation framework is to effectively transmit this pre-existing knowledge, rather than to generate new knowledge through the specific design.

### 2.6. Architectural Adjustment Studies

After a standard KD model was established as the primary method for feature representation transfer, a series of experiments were conducted. These experiments aimed to assess potential performance enhancements from common architectural modifications to the student model, instead of introducing new components as primary contributions. These modifications included the integration of lightweight attention modules and the utilization of advanced bounding box regression losses.

#### 2.6.1. Bounding Box Regression Loss Evaluation

In this experiment, the impact of substituting the default CIoU was tested with alternative bounding box regression functions on the localization precision of the distilled student model. Rose black spot lesions in field images exhibit irregular shapes and significant size variations. Given this characteristic, a loss function more attuned to diverse spatial attributes may improve detection performance. To empirically evaluate this hypothesis, six IoU-based variants were selected. These variants include CIoU (default), DIoU, SIoU, EIoU, GIoU, and Wise-IoU (WIoU), which were compared within distilled student model.

#### 2.6.2. Lightweight Attention Mechanism Integration

The effects of incorporating lightweight attention modules were also explored. The objective was to evaluate whether such modules could enhance the distilled student model’s capacity to concentrate on distinctive disease characteristics within intricate field environments. Six common attention mechanisms were chosen for this study, which are SE, CBAM, ECA, RFA, MLCA and SimAM. These modules were inserted following the C3k2 blocks in the feature extraction pathway. All integrations were designed to incur minimal computational overhead (≤0.2 GFLOPs increase) to maintain operational efficiency during deployment.

## 3. Results and Discussion

### 3.1. Knowledge Distillation Result

#### 3.1.1. Performance Overview

The performance of the three main models is illustrated in Table 2. The teacher model (YOLOv12-L) performed well on the selected lab data. The mAP@50 obtained in the teacher model (YOLOv12-L) was 98.8% with respect to the controlled samples. The result provided a good basis for knowledge transfer, as it shows that the teacher can extract disease-specific core features rather than noise from the environment. In contrast, the baseline student model (YOLOv12-N) was 2.56M parameters and 6.3 GFLOPs. It was trained only on the limited field dataset and achieved an mAP@50 of 77.6%. This performance highlights the challenges of field conditions and the domain shift impedes direct application of lab-trained models. However, the standard knowledge distillation framework significantly reduced this gap. The mAP@50 scores of the student model improved by 3.5% to 81.1%. The student model maintains a compact design, which is critical for resource-limited edge devices commonly used in rose cultivation such as handheld plant protection terminals for greenhouse inspections or low-altitude UAVs for open-field rose plantation patrols.

#### 3.1.2. Visualized Detection Improvements

**Figure 4:**
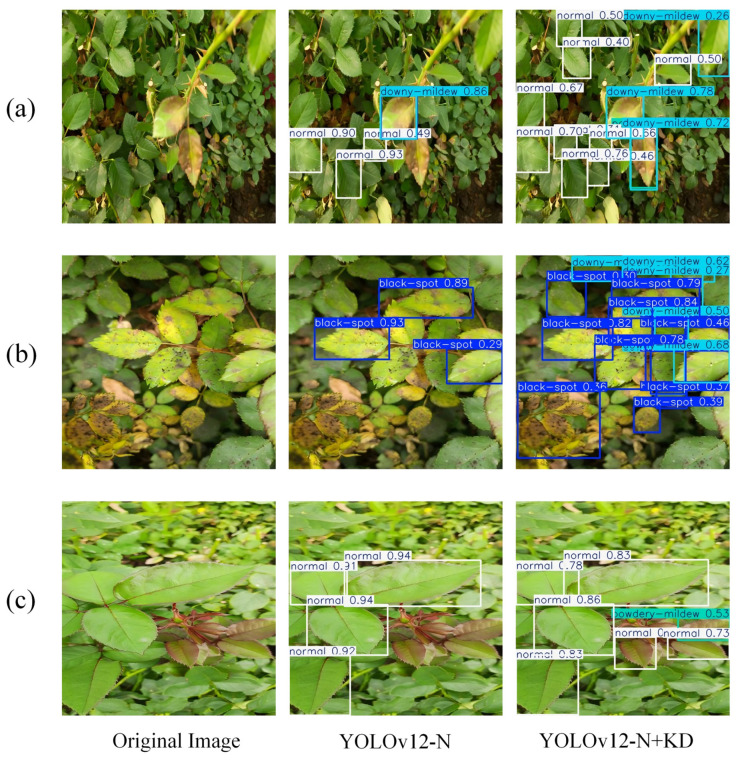
Visualized detection of rose leaf diseases. Different colored bounding boxes represent different types of plant diseases. (**a**) Detection performance on healthy leaves and downy mildew leaves. (**b**) Detection performance in multi-disease co-occurrence scenes. (**c**) Residual error case of the distilled model.

Qualitative improvements achieved through knowledge distillation are illustrated in Figure 4, which compares detection results between the baseline student and the distilled student across several representative field scenarios.

Figure 4a contains healthy leaves and leaves with downy mildew. The baseline student only identified the largest, centrally located diseased leaf and did not recognize adjacent smaller healthy leaves. The distilled model found many healthy leaves in the far edges of the leaf and erected bounding boxes that align more accurately with leaf margins. This suggests distillation improves the model’s tolerance to smaller and non-diseased foliage in a realistic field planting density.

Figure 4b presents a complex scene with co-occurring black spot and downy mildew. The baseline model detected only the most prominent black spot lesion near the center. The distilled model detected a downy mildew-affected leaf in upper-right corner and black spot leaf cluster in lower-left region, providing a consolidated bounding box around the affected area. Multi-disease scenarios are common in high humidity roses where downy mildew and black spot co-occur due to poor ventilation. The distilled model can improve its robustness in multi-disease scenarios. Nevertheless, fine-grained instance-level segmentation remains difficult.

Figure 4c shows a residual error mode of the distilled model. While the model correctly detected more healthy leaves, the model mistakenly classified one young yellow leaf as powdery mildew. This misjudgment stems from a natural physiological trait of rose young leaves, which exhibit slight yellowing during the unfolding stage; yellowing is different from white powdery mycelium characteristic of powdery mildew. This confusion is not only important for agricultural disease detection but also has practical implications for rose production, since misclassifying physiological traits as diseases can lead to unnecessary pesticide and chemical fertilizer application [8]. This can be due to insufficient samples of young rose leaves with natural yellowing in teacher model pretraining data.

Together, these comparisons show that the distilled light model achieved tangible gains for field disease detection. It expanded detection coverage to smaller, overlapping, or co-occurring lesions, and the localization accuracy improved for diseased and healthy plants. Although some edge cases were confused due to ambiguous foliage characteristics, the model made more sense for the variability and clutter of real-life agriculture settings.

#### 3.1.3. Grad-CAM Attention Map Analysis

**Figure 5:**
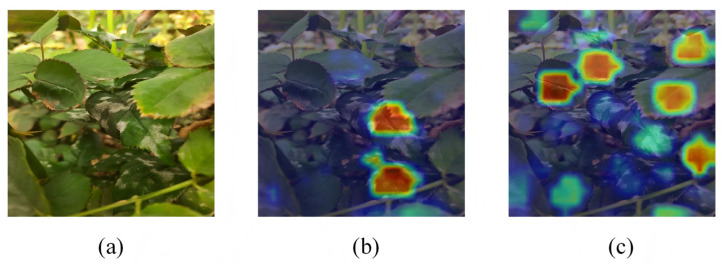
Grad-CAM attention maps. Darker and warmer colors like red and yellow indicate higher attention weights. (**a**) Image to be tested. (**b**) Baseline student model. (**c**) KD student model.

To illustrate the qualitative improvements in detection performance, Grad-CAM attention maps were used to visualize the model decision focus. YOLO relies on convolutional backbones, and Grad-CAM generates spatial attention heatmaps by fusing gradients and feature maps from a target layer. As a widely used tool for YOLO family models, Grad-CAM effectively distinguishes differences in attention focus between disease related regions and background noise. Figure 5 shows the attention distributions of both the baseline student and distilled student on representative field images of powdery mildew and downy mildew leaves in weed.

The attention map of the baseline student concentrated more on shadowed areas created by occluded leaves. This resulted in a higher false positive rate and lower precision, because the model allocated more resources to ambiguous or irrelevant elements. This distribution of attention is particularly problematic in real fields where variable lighting can produce misleading visual indications. The attention map of the distilled student concentrated more on the actual diseased areas, especially upper leaf areas where symptoms are more intense. This emphasis is due to the teacher model-prioritized disease-specific features acquired in controlled laboratory environments. This focus allows the lightweight model to filter environmental noise and focus on biologically relevant disease features. It also proves that knowledge distillation transfers the teacher’s discriminative knowledge to the lightweight student, which improve detection performance and decision reliability in precision agriculture applications.

#### 3.1.4. Robustness of Standard Distillation

Extensive hyperparameter tuning experiments were conducted to investigate the ability of the standard distillation framework to adjust to hyperparameters. The mAP@50 remained consistent at 81.1% across all tested hyperparameter combinations, and key detection metrics including Precision, Recall, and F1-score also showed no variations. The tested combinations covered variations in the temperature parameter τ=2,4,6 and adjustments to the weighting coefficients of the three distillation loss components, including $\lambda_{\mathrm{cls}}:\lambda_{\mathrm{box}}:\lambda_{\mathrm{obj}}=1:1:1$. Even with the removal of the L_box distillation loss component, the results remained unchanged. Two explanations are proposed. First, the student may have reached a local optimum under native detection loss, where distillation is marginally useful. Second, the gradient contribution of L_box may be small relative to native CIoU loss, making its effect statistically smaller for joint optimization. Crucially, this does not imply that localization knowledge is unimportant. Rather, explicit Localization Distillation provides no extra gain beyond what is already captured by the student’s native regression objective or implicitly transferred by teacher features. Future work should employ feature alignment or gradient analysis to investigate how localization information is implicitly encoded. From a deployment perspective, this stability is particularly valuable as it eliminates the need for complex hyperparameter tuning. This enables the model to adapt to various rose cultivation environments in southern China, including solar greenhouses and open fields with substantial variations in temperature, humidity, light, and rainfall.

However, this robustness challenges the traditional view of distillation research, which focuses on designing complex distillation methods and finely balancing multi-component losses. Thus, a major hypothesis is that for student models the maximum cross-domain adaptation performance depends more on the content distilled than on the distillation itself. This content is the quality of feature representations learned by the teacher model.

### 3.2. Pseudo-Labeling Experiment Result

To validate the hypothesis, a decisive ablation experiment was designed. A lightweight student model was trained in its final stage. Only hard pseudo-labels of the frozen teacher on the field training set were used for supervision. Experimental results between the pseudo-label student and standard KD student are presented in Table 3.

The pseudo-label student scored 80.8% of mAP@50 compared to the standard KD student (81.1%), whereas this 0.3% difference fell within the typical training variance margin. In addition to the core mAP@50, precision, recall, and F1-score also validated the equivalent performance between the two models. The pseudo-label student scored 79.5% precision, which is 0.4% higher than standard KD student. This improvement might have been related to filtering low confidence, uncertain predictions through hard labels corresponding to the teacher’s binary decisions. The KD model reported 70.3% recall and exceeded the pseudo-label student by 0.8%. This could have been due to probabilistic information in soft labels that can help when learning smoother decision boundaries for more challenging samples. The difference in their F1-scores was 0.3% and these slight variations did not affect overall detection performance.

**Figure 6:**
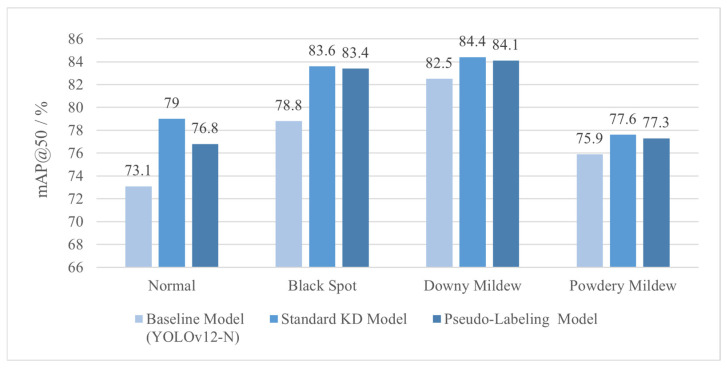
Per-class mAP@50 comparison between standard KD and pseudo-label model.

This similarity was consistent across all disease categories as shown in Figure 6. It suggested that both models inherited identical strengths and weaknesses from the teacher. Attention analysis might explain the slightly larger performance gap of 2.2% mAP@50 for the healthy leaf category. Soft label supervision by the standard KD student helped distinguish more nuanced boundaries between healthy tissue and field background. In contrast, the pseudo-label student’s hard labels missed this probabilistic guidance. Even though there was a slight advantage, the performance on other diseases also suggested that soft labels offered limited additional benefits over hard pseudo-labels.

This near-equivalence confirms the main argument of this paper. The essential knowledge needed for cross-domain performance enhancement is contained in the teacher’s output decisions on target domain data. The standard distillation with soft probabilities and additional losses does not produce qualitatively superior knowledge, but offers a better and more stable way of passing this same knowledge. The key difficulty for detecting rose leaf disease in a laboratory-field shift is the quality and generalizability of feature representations learned by the pre-trained teacher model. This problem arises because the teacher may not have been exposed to many real-world conditions in farming. For example, it may not have had images of rose diseases captured in rainy conditions where lesions are obscured by water droplets. Even with the best distillation process, such gaps in pre-trained data cannot be remedied. This concludes a direction for light cross-domain detection models for precision agriculture. Future studies should pay special attention to the feature expression of the teacher model.

### 3.3. Architectural and Loss Function Modifications

It was investigated whether typical architectural improvements could enhance performance beyond the limits set by standard distillation. Table 4 details the impact of different bounding box regression losses. Table 5 presents the outcomes of incorporating lightweight attention modules.

Unexpectedly, none of the modifications improved mAP@50 performance. Other bounding box regression losses either matched the performance of the default CIoU or underperformed compared to it. Attention mechanisms reduced mAP@50 by 5.9% to 8.0%, and increased computational demands, like RFA and MLCA, thereby compromising deployment feasibility. This followed the results of Section 3.1.4 and Section 3.2, which showed that once the student model assimilated the teacher’s feature knowledge through standard processes, its performance stabilized at a level inherent to that knowledge. Improvements to the student model or loss function resulted in diminishing or even negative returns.

This finding is important for lightweight field models in that overly complex architectures may weaken feature representation in agriculture scenarios. Real rose field images contain noise such as irrelevant leaf veins, soil spots, or dew marks. Complex modules like attention mechanisms may overfit these noise features instead of focusing on disease symptoms.

### 3.4. Benchmark Comparison

A benchmark comparison of mainstream detectors is presented in Table 6. To ensure fair comparison, all mainstream detectors in the benchmark were retrained on the same augmented field dataset under the unified training protocol described in Section 2.2.2. The distilled YOLO12-N had the highest mAP@50 at 81.1%, and achieved a precision of 79.1% and a recall of 70.3%. In addition, the model achieved an F1-score of 74.4%, which is the best of all detectors. The distilled model retained the same computational complexity as the baseline YOLO12-N, with a GFLOPs value of 6.3 and a parameter count of 2.56M. These metrics were on par with the most lightweight models, such as YOLOv10-N (2.27M parameters, 6.5 GFLOPs) and YOLOv5-N (2.50M parameters, 7.1 GFLOPs), while being much smaller than the larger models like YOLOv8-S (11.13M parameters, 28.4 GFLOPs) and YOLOv9-S (7.17M parameters, 26.7 GFLOPs).

The benchmark positions the distilled model as a leading performer among lightweight detectors for agricultural field deployment. The high accuracy and low computational cost of the model make it ideal for real-world applications, bridging the gap between algorithm performance and practical deployment. The model has an inference speed of 212.8 FPS, which is 27.7% faster than YOLO12-N and far faster than the real-time requirement of 15–20 frames per second for UAV patrols [25]. With 2.56M parameters and low computational overhead, it is easily deployed on low-cost devices for small- and medium-sized rose growers without high-performance computing resources.

### 3.5. Limitations and Future Work

This study has several limitations. First, the teacher model was trained on a public laboratory dataset, with limited learned representations, and it was lacking in samples from key production regions such as Yunnan. Second, pseudo-labeling may propagate errors from the teacher model, particularly for underrepresented rare disease symptoms. An example is the misclassification of young rose foliage with natural physiological yellowing as powdery mildew; this confusion between nonpathogenic plant traits and real disease symptoms can create practical challenges for field crop management.

To address these limitations and advance framework, several future research directions are proposed. Enriching pre-training datasets with samples from major rose-producing regions across varying environmental conditions will improve model robustness and generalizability. Second, active learning strategies such as uncertainty sampling will reduce error propagation in pseudo-labeling by prioritizing expert annotation of challenging samples. In addition, hardware-specific optimization is critical for real-world field deployment. Combining the framework with model compression techniques such as quantization and pruning will further reduce inference latency and enhance compatibility with low-power edge devices. Finally, validating the teacher–student framework on other agricultural vision tasks such as crop growth stage monitoring and yield estimation will further validate its potential as a flexible paradigm for lightweight AI solutions in precision agriculture.

## 4. Conclusions

This research addressed the critical challenge of balancing detection accuracy and computational efficiency for in-field rose leaf disease detection. The findings confirmed that for lightweight rose disease detection, the quality of transferable feature representations from a pre-trained teacher model (YOLOv12-L) was the primary driver of performance gains in field environments, rather than the complexity of the distillation architecture itself.

Three key insights emerged with practical implications. First, the nearly identical performance of standard knowledge distillation and simple pseudo-label training suggested that the main benefit transferred was the robust disease-specific feature discriminability of the teacher rather than the specific format of the supervisory signal. Second, the standard distillation framework was robust and not sensitive to key hyperparameters such as temperature and loss weighting. This made tuning easy for end users. Third, maintaining simplicity of the student model was beneficial. Attempts to enhance the distillation student with additional modules like attention mechanisms or other loss functions often yielded weak or negative returns, suggesting that unnecessary complexity could reduce edge deployment efficiency.

Guided by these insights, the distilled student model achieved an optimal accuracy-to-efficiency ratio. It attained an mAP@50 of 81.1%, while maintaining a computational footprint of 2.56 million parameters and 6.3 GFLOPs. This performance profile made it well-suited for deployment on resource-constrained devices, such as low-altitude UAVs or handheld protection terminals. Qualitative analysis further confirmed that the proposed framework improved detection coverage for smaller, overlapping, or co-occurring lesions, effectively filtering environmental noise such as leaf shadows and soil patterns through the transfer of discriminative knowledge.

In summary, this study shifted the focus from designing complex distillation mechanisms to leveraging high-quality teacher representations, providing a practical and effective framework for bridging the lab-to-field domain gap. The observed robustness of the model across various hyperparameter configurations suggests a high potential for scalability across the heterogeneous microclimates of solar greenhouses and open fields in Yunnan Province.

## Figures and Tables

**Table 1:** Original dataset specifications.

Dataset	Total Images (Original)	Resolution	Train/Val/Test Split (Original)
Laboratory Dataset	2000	1024 × 1024	1400/300/300
Field Dataset	990	416 × 416	792/99/99

**Table 2:** Performance of core models.

Model	P/%	R/%	mAP@50/%	mAP@50-95/%	F1-Score/%	GFLOPs	Parameters	Layers	FPS
YOLOv12-L	98.2	95.0	98.8	79.5	96.6	88.6	26.34M	283	112.4
YOLOv12-N	76.2	69.8	77.6	58.0	72.9	6.3	2.56M	159	166.7
YOLOv12-N + KD	79.1	70.3	81.1	58.8	74.4	6.3	2.56M	159	212.8

**Table 3:** Standard KD vs. pseudo-label training.

Model	P/%	R/%	mAP@50/%	mAP@50-95/%	F1-Score/%
YOLOv12-N + KD (Soft Label)	79.1	70.3	81.1	58.8	74.4
YOLOv12-N + KD (Hard Label)	79.5	69.5	80.8	58.8	74.1

**Table 4:** Performance with alternative regression losses.

Loss Function	P/%	R/%	mAP@50/%	mAP@50-95/%	F1-Score/%	GFLOPs	Parameters	Layers	FPS
CIoU (default)	79.1	70.3	81.1	58.8	74.4	6.3	2.56M	159	212.8
DIoU	79.8	68.9	78.3	59.1	74.0	6.3	2.56M	159	217.4
SIoU	71.1	75.1	77.8	57.0	73.0	6.3	2.56M	159	104.2
EIoU	84.9	66.3	76.9	58.4	74.5	6.3	2.56M	159	588.2
GIoU	77.6	69.1	79.9	58.7	73.1	6.3	2.56M	159	137.0
WIoU	74.7	67.4	76.0	57.7	70.9	6.3	2.56M	159	80

**Table 5:** Performance with integrated attention modules.

Attention Module	P/%	R/%	mAP@50/%	mAP@50-95/%	F1-Score/%	GFLOPs	Parameters	Layers	FPS
YOLOv12-N + KD	79.1	70.3	81.1	58.8	74.4	6.3	2.56M	159	212.8
SE	68.8	67.9	73.7	52.3	68.3	6.3	2.57M	174	217.4
CBAM	69.8	71.2	75.2	52.3	70.5	6.4	2.57M	183	158.7
ECA	69.8	67.2	73.1	52.0	68.5	6.3	2.56M	168	158.7
RFA	76.2	62.3	73.6	52.4	68.9	6.5	2.58M	183	111.1
MLCA	71.4	64.6	73.5	52.2	67.8	6.4	2.63M	192	65.8
SimAM	74.0	65.9	74.2	52.8	69.7	6.3	2.56M	162	100

**Table 6:** Comparison with mainstream detectors.

Model	P/%	R/%	mAP@50/%	mAP@50-95/%	F1-Score/%	GFLOPs	Parameters	Layers	FPS
YOLOv5-N	72.8	70.8	75.7	56.5	71.8	7.1	2.50M	84	384.6
YOLOv8-N	72.1	69.7	76.1	57.3	70.9	8.1	3.01M	72	92.6
YOLOv8-S	78.3	67.4	76.8	57.9	72.4	28.4	11.13M	72	208.3
YOLOv9-S	77.1	68.6	78.1	59.9	72.6	26.7	7.17M	197	322.6
YOLOv10-N	78.5	66.6	77.0	58.4	72.1	6.5	2.27M	102	666.7
YOLOv10-S	75.6	69.0	78.0	59.0	72.1	21.4	7.22M	106	434.8
YOLO11-N	76.7	70.2	76.9	58.2	73.3	6.3	2.58M	100	243.9
YOLO12-N	76.2	69.8	77.6	58.0	72.9	6.3	2.56M	159	166.7
YOLO12-N + KD	79.1	70.3	81.1	58.8	74.4	6.3	2.56M	159	212.8

## Data Availability

The data used in this study are derived from public domain resources and are accessible at the following links: Cut-flower Disease Dataset, available at https://data.mendeley.com/datasets/nmjd35fcb5/2 (accessed on 12 October 2025); Multifaceted Rose Leaf Disease Dataset for AI-Driven Plant Pathology, available at https://data.mendeley.com/datasets/8jtfk9szbg/3 (accessed on 12 October 2025).

## Abbreviations

The following abbreviations are used in this manuscript:

KD	Knowledge Distillation
CIoU	Complete Intersection over Union
DIoU	Distance Intersection over Union
SIoU	Spatial Intersection over Union
EIoU	Enhanced Intersection over Union
GIoU	Generalized Intersection over Union
WIoU	Weighted Intersection over Union
SE	Squeeze-and-Excitation
CBAM	Convolutional Block Attention Module
ECA	Efficient Channel Attention
RFA	Rotated Feature Attention
MLCA	Multi-scale Local Channel Attention
SimAM	Simple Attention Module
